# Supplementary material for: Submersible touchless interactivity in conformable textiles enabled by highly selective overbraided magnetoresistive sensors
Source: Commun Eng. 2025 Feb 25;4:33. doi: 10.1038/s44172-025-00373-x (PMC11861257; doi:10.1038/s44172-025-00373-x)
Supplement: Supplementary file 2 — Supporting information [file 44172_2025_373_MOESM2_ESM.pdf]

Submersible touchless interactivity in conformable textiles enabled by highly selective overbraided magnetoresistive sensors

Pasindu Lugoda[1*,#], Eduardo Sergio Oliveros-Mata[2,#], Kalana Marasinghe[3], Rahul Bhaumik[4], Niccolò Pretto[4], Carlos Oliveira[3], Tilak Dias[3], Theodore Hughes-Riley[3*], Michael Haller[4], Niko Münzenrieder[4*], and Denys Makarov[2*]

[1] Department of Engineering, School of Science and Technology, Nottingham Trent University, Nottingham, U.K.

[2] Helmholtz-Zentrum Dresden-Rossendorf e.V., Institute of Ion Beam Physics and Materials Research, 01328 Dresden, Germany

[3] Advanced Textiles Research Group, Nottingham School of Art and Design, Nottingham Trent University, Nottingham, U.K.

[4] Faculty of Engineering, Free University of Bozen-Bolzano, Bozen-Bolzano, Italy

Supplementary Tables

Supplementary Table S1. Comparison of magnetic sensing technologies for electronic textiles, highlighting the successful implementation of magnetic field sensing at the braided level, enabling conformability and performance in knitted fabrics.

Ref	Sensor Type	Integration Method	Sensitivity/LOD	Sensor Form Factor
[1]	Magnetic Eddy Current Induction	Coils sewed into a shirt	Not specified	Wire (coil)
[2]	Hall Effect	Integrated with conductive woven fabric	Not specified	Rigid (ESP32 module)
[3]	Dual-mode: Magneto-straining	Coating on spandex substrate	0.7 kPa^{-1} (pressure), 13.76 mm^{-1} (proximity)	2D coating Film
[4]	Dual-mode: Strain & Magnetic Field	Dropping-drying process	44% resistance change at 8% strain, 1.5% relative resistance increment at 44° bending under 400 mT	1D Coaxial structure
This work	Giant Magnetoresistive (GMR)	Overbraiding of a flexible thin film sensor	380 nT, > 7% relative resistance increment at 450 mT	1D overbraided sensor

Supplementary Table S2. Comparison of flexible magnetic sensor technologies for interactive applications. While previous works have focused on thin-film or printed sensor formats, this work introduces an approach utilizing a 1D overbraid form factor for GMR sensors. This strategy results in highly sensitive and comfortable interactive textiles, enabling seamless integration of magnetic sensing into fabrics.

Ref	Sensor Type	Sensor Form Factor	Integration Method	Sensitivity/LOD
[13]	Magnetoelectric (ME)	Flexible Laminate	Bonding of Metglas foils to PZT thick film on mica substrate	200 nT at low frequencies, 200 pT at resonance
[14]	Hall Effect	Flexible (Laser-Scribed Graphene)	Laser scribing on polyimide substrate	1.12 V/AT, 0.446 mT/Hz resolution
[15]	Anisotropic Magnetoresistive (AMR)	3D Micro-Origami Cubic Architecture	Self-assembly of micro-origami cubes on a wafer-scale, integrated with a-IGZO TFTs	(0.068 ± 0.022) T^{-1} for three orthogonal axes
[16]	Anisotropic Magnetoresistive (AMR)	Printed, Flexible	Printed onto flexible substrates	35.7 T^{-1} at 86nT, 36 nT resolution
[17]	Anisotropic Magnetoresistive (AMR)	Flexible	Deposited on ultrathin Kapton substrate	0.25 Oe^{-1}
[18]	Giant Magnetoresistive (GMR)	Printed, Flexible	Printed on ultrathin polymer foils	3 T^{-1} at 0.88 mT
This work	Giant Magnetoresistive (GMR)	1D overbraided sensor	Overbraiding of a flexible thin film sensor	380 nT resolution, >7% relative resistance increment at 450 mT

Supplementary Note 1: Process of crafting overbraided magnetoresistive sensors

This section explains the procedure for crafting overbraided magnetoresistive sensors, detailing the rationale behind the material and method selection. It will demonstrate the methodology used to attach the thin film sensor, followed by the encapsulation and braiding steps utilized to provide mechanical protection for both the sensor and its interconnects. The performance of GMR sensor prepared on different polymeric foils is summarized in Supplementary Figure S1. Supplementary Figure S2 demonstrates the sensor's excellent stability after repeated cyclic bending. Supplementary Figure S3 compares the performance of the Co/Cu-based GMR multilayers deposited on different substrates. The homogeneity of the Co/Cu deposition obtained through profilometer measurements using a Dektak device is presented in Supplementary Figure S4.

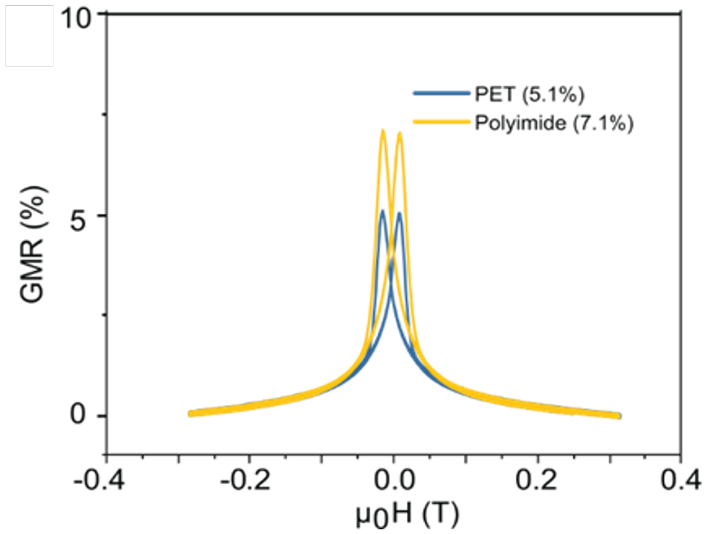

Supplementary Figure S1. Performance of flexible GMR sensors. The magnitude of the magnetoresistance effect of GMR samples fabricated on different polymeric foils sputtered under identical conditions. (a) [Co/Cu] GMR stacks coupled at the 2nd antiferromagnetic maximum showed a GMR effect of about 5.1% and 7.1% when grown on PET and polyimide substrates, respectively. The polyimide flexible substrates are resilient to processing and showed increased magnetoresistance against PET substrates. We fabricated the overbraided magnetoresistive sensors using sensors fabricated on polyimide foils.

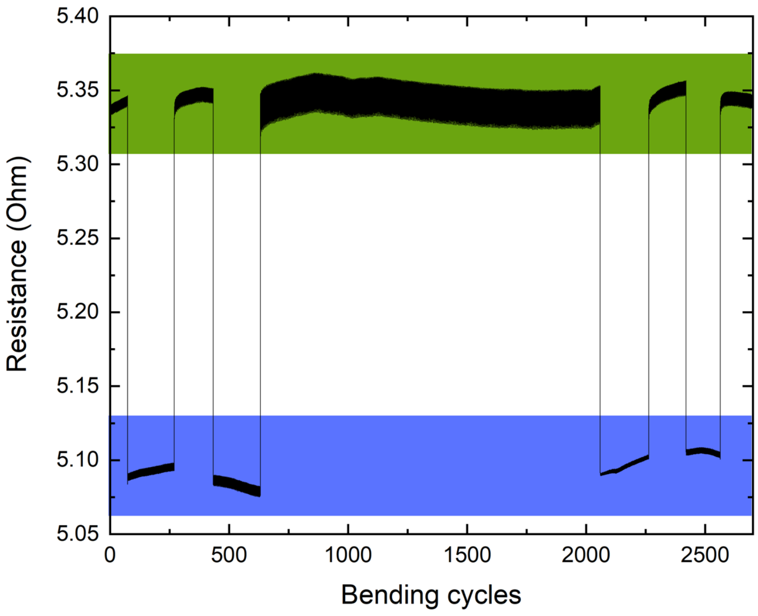

Supplementary Figure S2. **Dynamic bending test of flexible GMR sensors.** Thin-film GMR sensors exhibit negligible performance degradation under dynamic cyclic bending. The green shadowed area shows the resistance values during bending cycles without field and the blue shadowed area corresponds to the resistance value of the GMR sensor when a magnet (100 mT) is approached during the experiment.

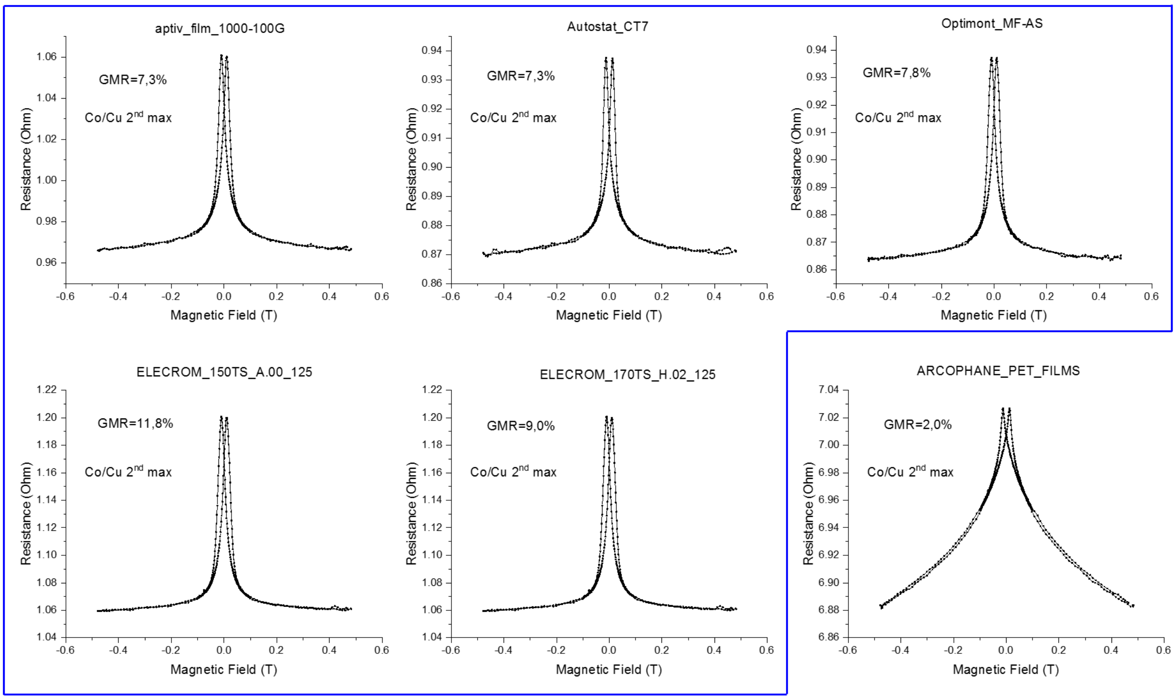

Supplementary Figure S3. GMR performance of sensors on different polymeric foils. Comparison of the magnetoresistive performance of Co/Cu-based GMR multilayers deposited on different flexible substrates. The blue box indicates sensors, which reveal the GMR performance comparable to a reference sensor prepared on a rigid SiO_2/Si wafer.

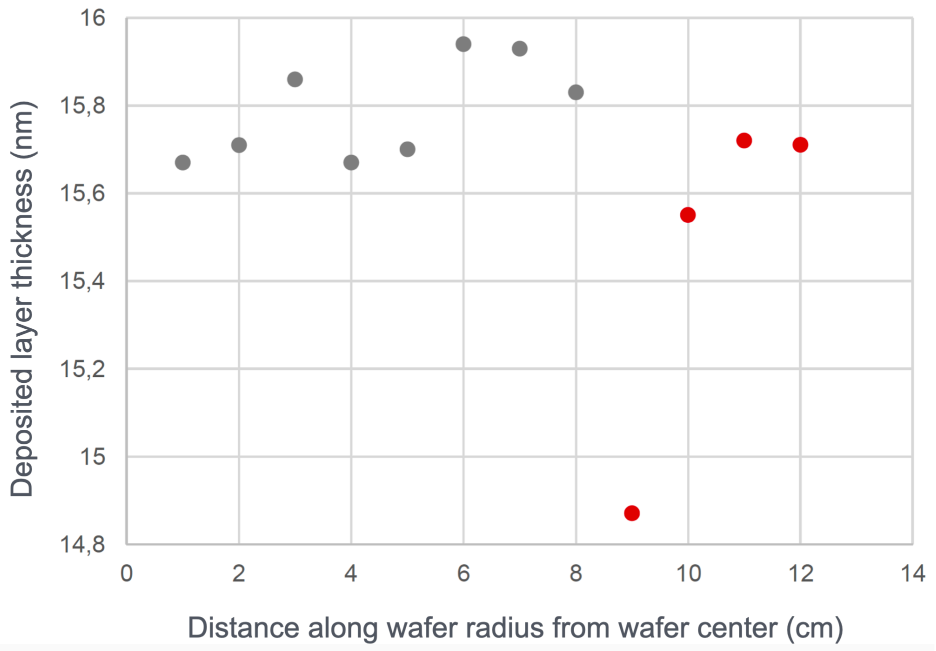

Supplementary Figure S4. Homogeneity of the deposited Co layer. Homogeneity of the deposited Co layer measured by using a Dektak profilometer. Gray symbols correspond to the measurements carried out of the samples prepared within the inner part of a wafer (radius is smaller than 90 mm). Red symbols correspond to the measurements carried out of the samples prepared within the outer part of a wafer (radius is larger than 90 mm).

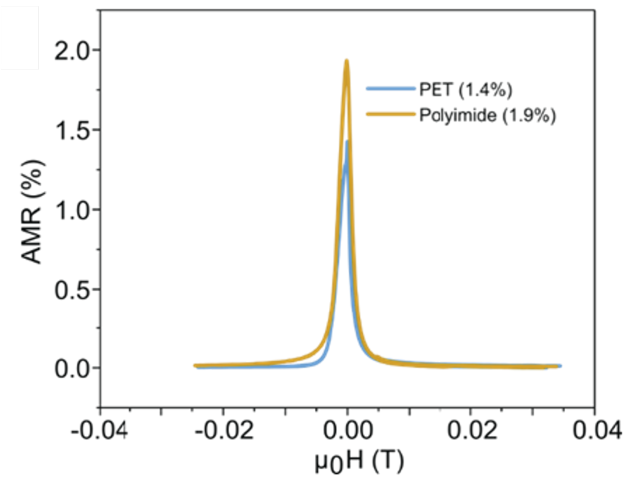

Supplementary Figure S5. Performance of flexible AMR sensors. The magnitude of the magnetoresistance effect of AMR samples fabricated on different polymeric foils sputtered under identical conditions. Permalloy-based AMR films showed an AMR effect of about 1.4% and 1.9% when grown on PET and polyimide substrates, respectively.

The schematic representation of the fabrication steps of the overbraided magnetoresistive sensor utilizing the thin-film magnetoresistive sensors are illustrated in Supplementary Figure S6.

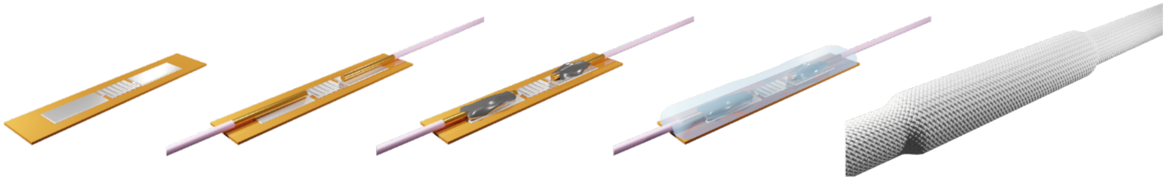

Supplementary Figure S6. Schematics of the fabrication process. A schematic representation of the fabrication process of a overbraided magnetoresistive sensor. A litz wire is positioned on contact pads of a magnetoresistive sensor prepared on polymeric foil. Then the sensor is attached to the litz wires using a conductive epoxy. Thereafter, the sensor with the interconnects is encapsulated using an epoxy resin and the encapsulated structure is positioned within a textile braid.

1.1 Attaching the sensor to conductive wires

The sensor utilized for overbraided magnetoresistive sensors contains either a GMR [Cu/Co] thin film stack or permalloy-based AMR layers deposited onto a polyimide foil of 50 µm thick. The sensor comprises two terminals that can be utilized for acquiring resistance measurements. When the sensors are positioned within a textile braid, a key requirement is having sufficiently long electrical connection to the interface electronics or the sensor conditioning circuit. Several criteria must be fulfilled to ensure electrical connectivity within the textile braid:

I. Low resistance: The resistance of the conductive wire/path must be sufficiently low >200 Ω/m to not affect the sensor measurements.

II. Short circuit prevention: Several of these overbraided magnetoresistive sensors can be used in a textile. Hence, it is imperative to prevent short circuiting.

III. Protection from external factors: The connecting wires/paths must endure exposure to elements like water and sweat without resistance fluctuations.

IV. Endure textile deformations: Maintenance of resistance values upon mechanical deformations such as stretching, bending, and twisting.

1.1.1 Selection of connectors

This section gives an overview of the materials that can be used for the connection and reasoning for selecting litz wires for this work (BXL2001). Among the connectors deemed suitable for this application are stainless steel yarns, silver-coated conductive yarns, enamelled/bare copper wire (single core and multi-strand), printable conductive material and litz wires.

Metallic wires, including stainless steel and copper, exhibit low resistance values. However, they lack insulation, making them prone to short circuiting if positioned adjacent to other overbraided magnetoresistive sensors. Additionally, the presence of sweat and water, common in the environment where smart textiles are worn, can potentially affect the sensor measurements.[5] Another challenge arises from the differing mechanical characteristics of textiles and metal wires, leading to the protrusion of wires from the braid structure when the

textile undergoes deformations like bending or draping. This issue is also frequently encountered with enameled copper wires. Silver-coated conductive yarns exhibit textile-like behaviour. However, being a coated yarn with the conductive element exposed, leading to the same problems as indicated above for metallic wires. Moreover, silver-coated yarns corrode when exposed to sweat.[6] This can cause a break in the electrical path, disrupting the measurements. Printed conductive pathways, including ink-jet printed, and screen printed once demonstrate textile-like properties. Nonetheless, when the conductive pathways are deformed (because of washing or daily wear), the resistance of the conductive path varies, rendering this type of conductive pathway unsuitable for resistive measurements.[7,8]

The wire selected for the preparation of overbraided magnetoresistive sensors was a litz wire. Litz wires consist of multistrand copper wires where each strand is enamel coated before being covered together in polyester filaments. The litz wires chosen for this work feature the outer diameter of 254 µm,[9] resistivity of 1.329 ± 0.002 Ω/m (BXL2001). The enamel coating provides insulation for the copper, preventing any short circuiting and ensuring that the functionality remains unaffected in wet and sweaty conditions. The litz wire used in this work shows yarn-like properties and deforms similar to a textile without impacting the wires resistance.

1.1.2 Interconnection formation

Here, the focus lies on the formation of connections between thin film magnetoresistive sensors and litz wires. There are several approaches that can be taken for creating the interconnections including direct soldering, infrared soldering, direct current welding, mechanical fastening, and glue/bonding. The sensors were attached to the wire using conductive glue.

The subsequent section examines advantages and disadvantages of various interconnection methods and elucidates the rationale behind the selection of attachment using conductive glue. Soldering directly with a conventional soldering iron presents difficulties owing to the limited size of the contact area on the sensor. We experimented with the realisation of electronic sensing yarns using infrared soldering.[10] This technique exposes thin film sensors to high temperatures exceeding 220°C, potentially causing damage to the multilayer stack of the magnetoresistive sensors. Direct current closed-loop feedback resistance welders have been utilised to connect flexible electronic circuits with enamelled wires. However, this technique also causes heating of the contact pads. Hence, conductive glue emerged as the preferred choice for establishing the connection between the sensor and litz wires.

Conductive glue used in this work is a two component glue. Each of the components remains liquid at room temperature and solidifies only when mixed. The conductive glue was prepared in small batches to avoid wastage of material. The ends of a litz wire were heated using a conventional soldering iron and solder flux. The cleaned end of the litz wire was placed onto the designated contact pad on the sensor element. To ensure a reliable electrical connection, conductive glue was applied at the joining point. After the solidification of the interconnection, the wires were utilized to measure electrical resistance of the sensors. The sensor attached to the litz wires is shown in Supplementary Figure S7.

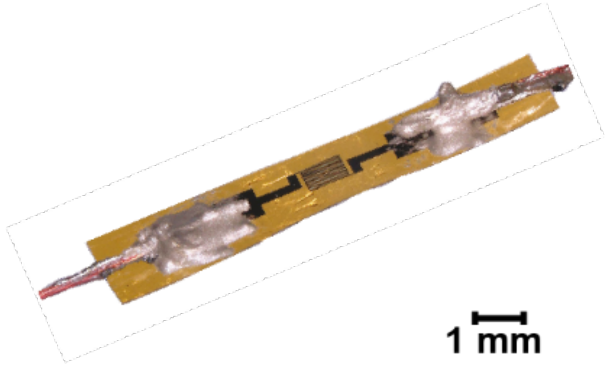

Supplementary Figure S7. Contacting a flexible magnetic field sensor. Optical micrograph showing the contacted sensor. Conductive glue is applied to connect litz wires to the sensor terminals.

1.2 Encapsulation

To enhance the sensor's durability, a protective layer should be applied to safeguard both the sensor surface and interconnects. This section provides information on methodologies that can be used to enhance mechanical integrity and protect the sensor from external elements. Introducing extra materials for sensor protection increases the resulting overbraided magnetoresistive sensor's diameter. Excessively increasing the braids dimensions impacts both the aesthetics of the textile and its wearability. Therefore, the list of requirements for the encapsulation step is as follows

I. Protection from external conditions.

II. Maintain small dimensions.

III. Enhance mechanical integrity.

Different techniques can be used for encapsulation.[11] One of these techniques is to use commercially available heat shrink tubes. While integrating these tubes is relatively simple, controlling the direction of deformation proved challenging, and it did not provide a complete coverage around the entire structure. One approach to achieving a more thorough coverage is by employing an ultraviolet (UV) curable polymer encapsulant. UV-curable encapsulants are favored over heat-curable ones due to the potential risk of sensor damage from heat.[11] Additionally, opting for a single-part encapsulant that cures upon exposure to UV light ensures quicker processing. The encapsulated sensors and interconnects are shown in Supplementary Figure S8.

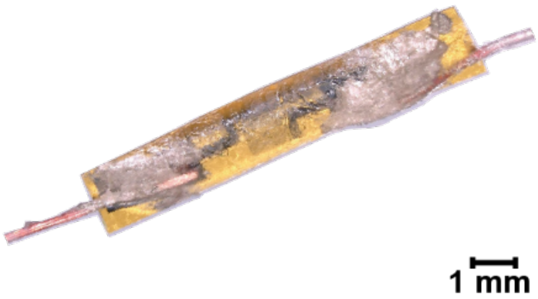

Supplementary Figure S8. Encapsulated magnetic field sensor. Optical micrograph shows the contacted sensor after encapsulation. UV cured resin is used to protect the sensor.

1.3 Textile cover

To render the electronics inconspicuous, it is necessary to cover the exposed encapsulated sensors and wires. This additional covering offers enhanced mechanical protection to sensors. The list of requirements for the covering stage is as follows.

I. The textile cover must be comprehensive, concealing the electronics within its layers.

II. It must provide mechanical support for the electronics.

The covering of flexible sensors can be conducted using different industrial braiding and covering machines. In past studies three different braiding techniques were explored for incorporating sensors (Supplementary Figure S9).[12] The decision to utilize a braiding machine (Supplementary Figure S9, right panel) for this application was made instead of opting for the double cover (Supplementary Figure S9, middle panel) and knit braid (Supplementary Figure S9, left panel) due to its ability to offer a more extensive coverage for flexible sensors. While the double cover technique produces structure with the smallest diameter compared to other methods, it still leaves areas of the sensors exposed. Therefore, braiding was preferred for the realization of overbraided magnetoresistive sensors. Experimental investigation identified a braiding lay length of 6 as optimal for covering the encapsulated sensor with polyester yarns. This lay length differs slightly from the lay length used for overbraided flexible temperature sensors,[12] where a lay length of 5 was employed.

To further improve the mechanical integrity of interconnects and to protect them from tensile forces, several textile polyester flat yarns were placed parallel to the sensor direction. These fibres were incorporated within the braid. This method ensures that the effects of tensile forces such as bending, twisting, and stretching on the overbraid sensor have minimal impact on the conductive wires, interconnections, and sensors embedded within it.

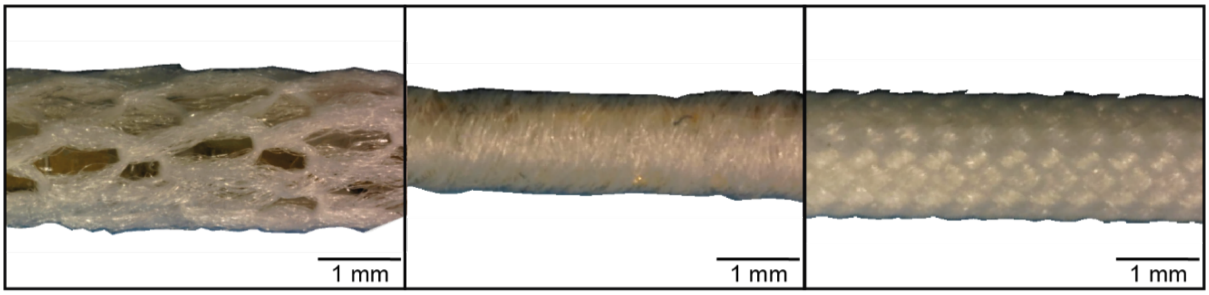

Supplementary Figure S9. Different textile covering techniques. (left) knit braid, (middle) double cover, (right) braid.[12]

Supplementary Note 2: The seamless jacquard sleeve structure

A jacquard sleeve was knitted using a Stoll CMS ADF 32W E7.2 machine, employing a ground tubular single jersey knit. Opting for a seamless 3 dimensional (3D) single jersey tubular knit structure facilitated the creation of the sleeve in one process, enhancing comfort without seams. The 3D sleeve structure is displayed in Supplementary Figure S10. Rib structures were knitted on both ends of the sleeve to ensure a snug fit around the arm. A tubular pocket was integrated to house the interface electronics, with a ribbed flap covering the pocket. Additionally, four 3D tubular channels were incorporated into the knitted sleeve to accommodate the four overbraided magnetoresistive sensors, ensuring seamless integration of all the electronics within the textile structure. While overbraided magnetoresistive sensors can be directly woven into fabrics, the decision to employ a knitted channel structure was deliberate. This choice allows for easier removal of the overbraided magnetoresistive sensors from the fabric. This ensures the separate recycling of the sensors and fabric components.

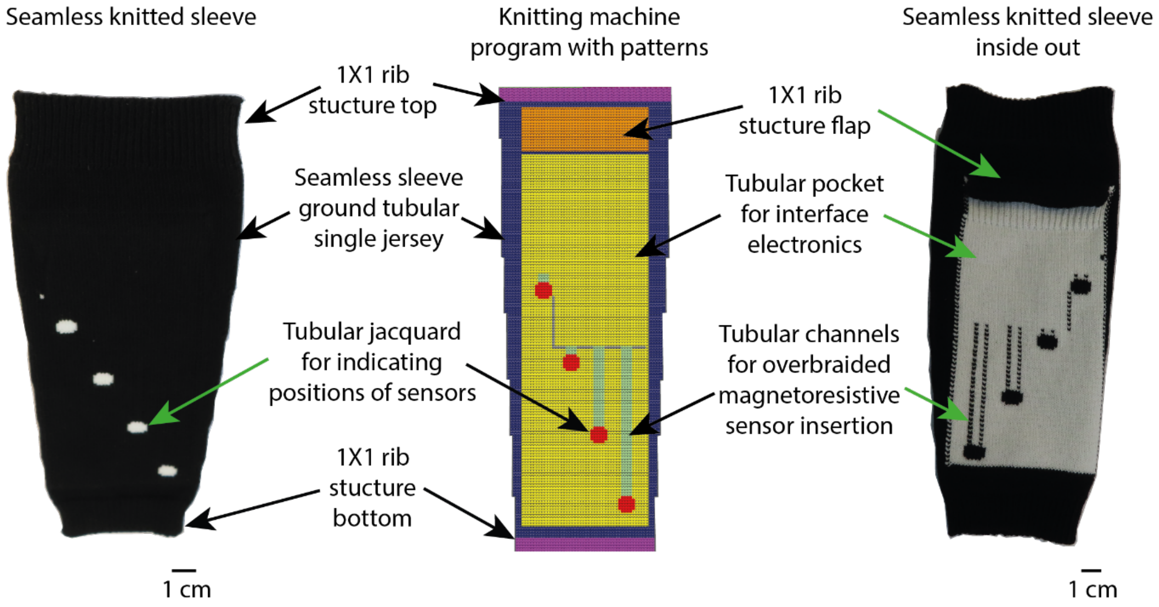

Supplementary Figure S10. Textile sleeve with embedded magnetic field sensors. A seamless sleeve embedded with overbraided magnetoresistive sensors along with the knit structure that was uploaded to the Stoll CMS ADF 32W E7.2 machine.

Supplementary Figure S11 demonstrates the textile sleeve containing the overbraided magnetoresistive sensors conforming around a mannequin forearm.

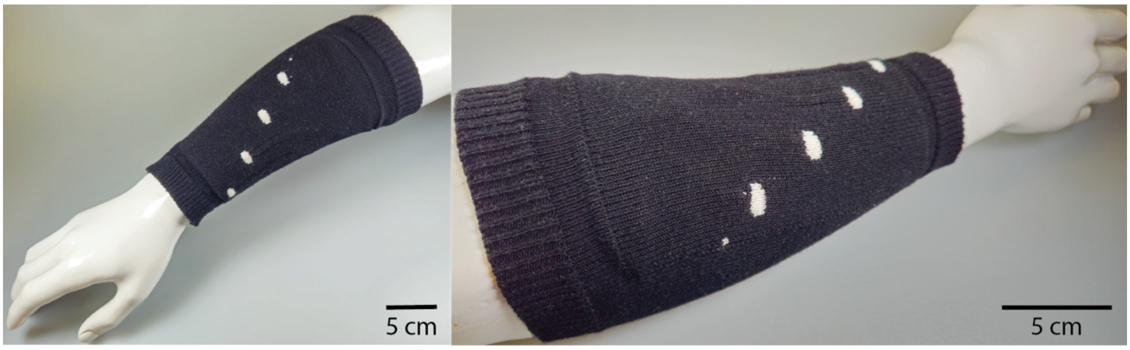

Supplementary Figure S11. Textile sleeve on a mannequin forearm. Images of the textile sleeve containing four overbraided magnetoresistive sensors worn on a mannequin forearm. The sleeve fits snugly and conforms to the shape of a mannequin forearm.

Supplementary Note 3: The seamless glove structure

A glove containing a N52 Neodymium magnet (N52 permanent magnet, diameter 3 mm and thickness 1 mm, surface magnetic field: 4105 G, pull force: 0.14 kg, Magnet Expert LTD, Newark, UK) was seamlessly knitted using a Stoll CMS ADF 32W E7.2 machine. A design preference for a 3D single jersey tubular knit structure was adopted for the glove. This choice allowed the glove to be knitted in a single process, ensuring comfort without the presence of seams. The 3D structure of the glove is shown in Supplementary Figure S12. Additionally, a tubular pocket was incorporated into the knit structure to accommodate the insertion of the magnet.

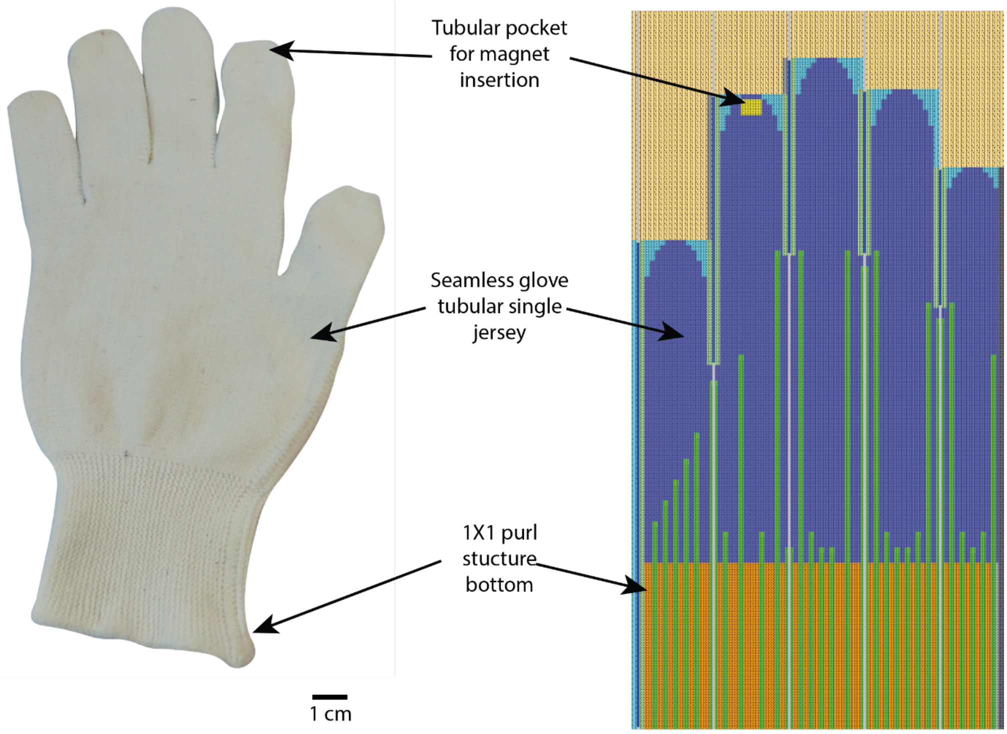

Supplementary Figure S12. Textile glove with embedded magnet. The seamless glove embedded with a miniature magnet and the knit structure that was uploaded to the Stoll CMS ADF 32W E7.2 machine.

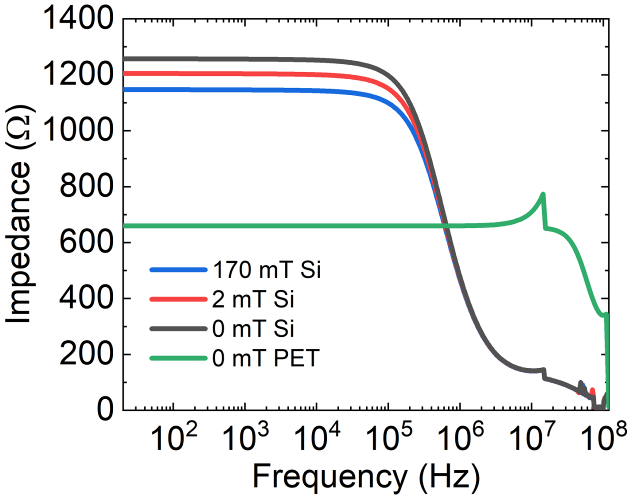

Supplementary Figure S13. Frequency response of GMR sensors. The change in electrical impedance of the GMR sensor as a function of frequency. The curves correspond to a sensor prepared on a rigid Si wafer and a sensor fabricated on a flexible foil. The impedance magnetic response remains stable up to 100 kHz, indicating a suitable bandwidth for real-time interactive applications.

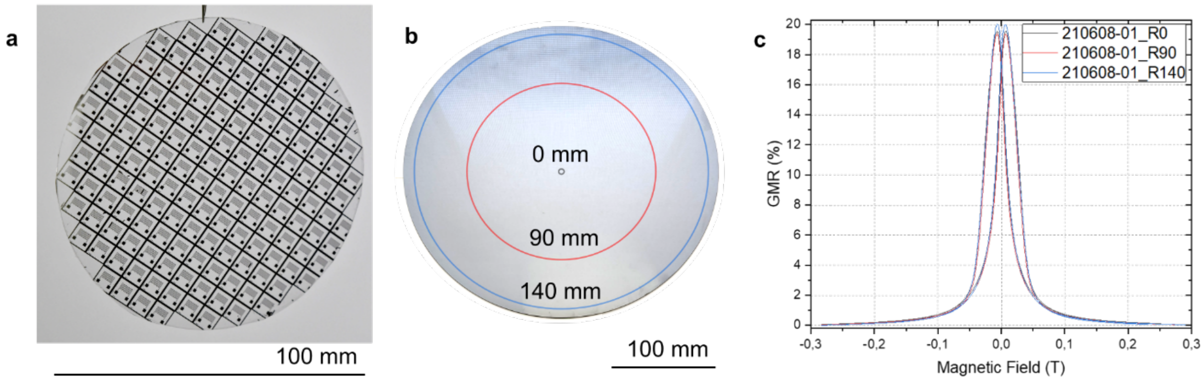

Supplementary Figure S14. Large area fabrication of flexible magnetic field sensors. High throughput fabrication of GMR sensors on large area polymeric foils. (a) Photograph of an array of >100 GMR sensors on a foil of 100 mm diameter. (b) A photograph of a 300 mm wafer deposited with GMR [Co/Cu] stacks fabricated via large area magnetron sputtering. The pieces are cut out at radii of 0 mm, 90 mm, and 140 mm for magnetoresistance characterization. The red circle corresponds to the radius where the GMR was measured at 90 mm. The blue circle corresponds to the radius where the GMR was measured at 140 mm. (c) GMR curves measured at different locations on the 300 mm substrate (indicated with circles in panel (b)). The high throughput, large area, and homogeneous fabrication of GMR sensors indicates the possibility to scale this technology for the required supply needed for distributed flexible sensors in textiles.

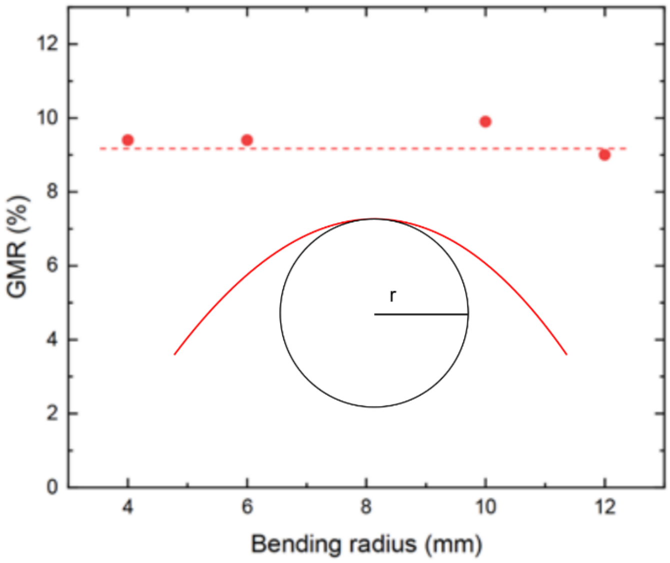

Supplementary Figure S15. Bending performance of flexible GMR sensors. Stability of the GMR sensor performance during bending deformations of samples fabricated on polyimide foils of 50 μm thick. Sensors fabricated on polyimide foils showed stable sensing performance under bending down to 4 mm radius. This results in a stable sensor response under mechanical deformations expected during wearing of a textile.

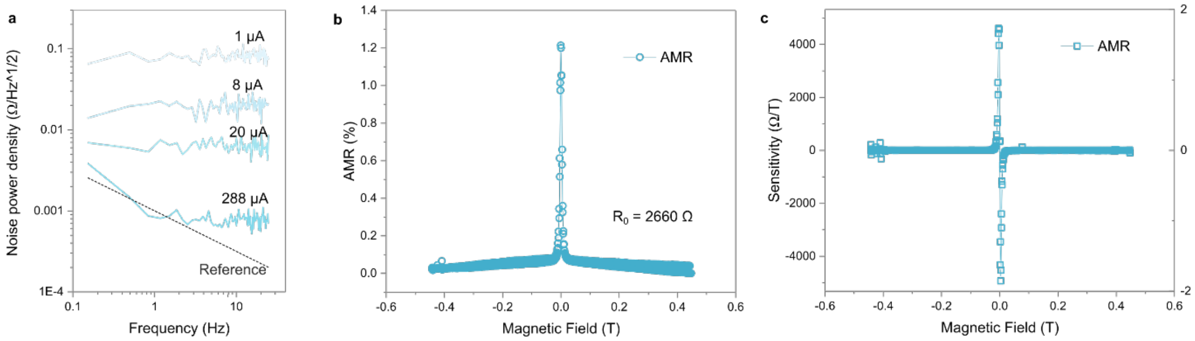

Supplementary Figure S16. Sensing performance of an AMR overbraided magnetoresistive sensor. (a) Resistive noise spectra of AMR overbraided sensors showing a 1/f noise after supplying the sensor with 288 μA (nominal sensor resistance is 2660 Ω). (b) The AMR sensor reveals good sensing performance in fields of below 50 mT with a high sensitivity of > 4000 Ω/T as shown in panel (c).

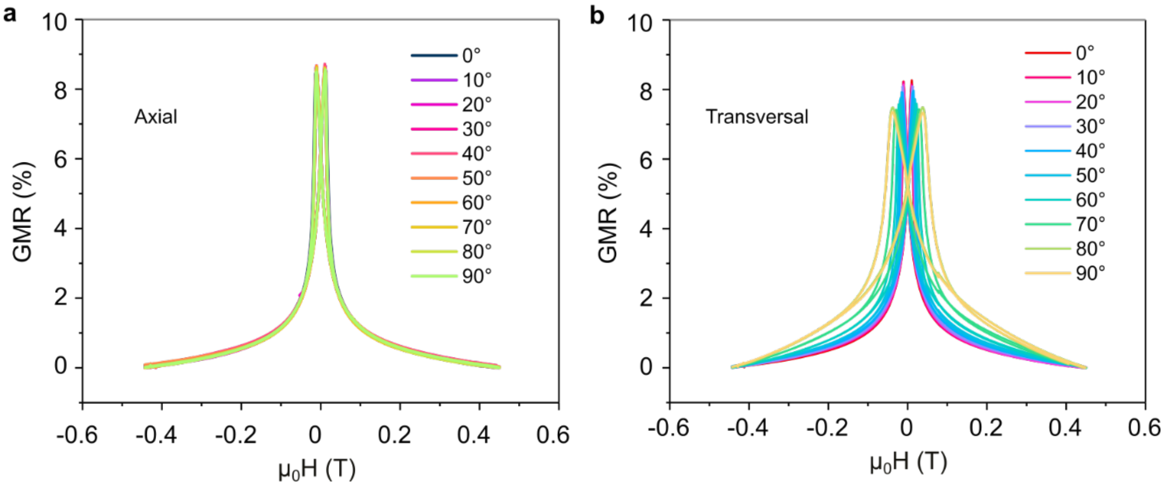

Supplementary Figure S17. Angular-dependent response of flexible GMR sensors. GMR responses of overbraided magnetoresistive sensors measured at different angular position of the sensor plane with respect to the magnetic field. GMR response of a sensor rotated (a) around the overbraided sensor's axis and (b) transversal to the overbraided sensor's axis. The GMR response shows an isotropic sensing for rotations around the axis while maintaining a full GMR response for rotations transversal to the direction of the overbraided sensor.

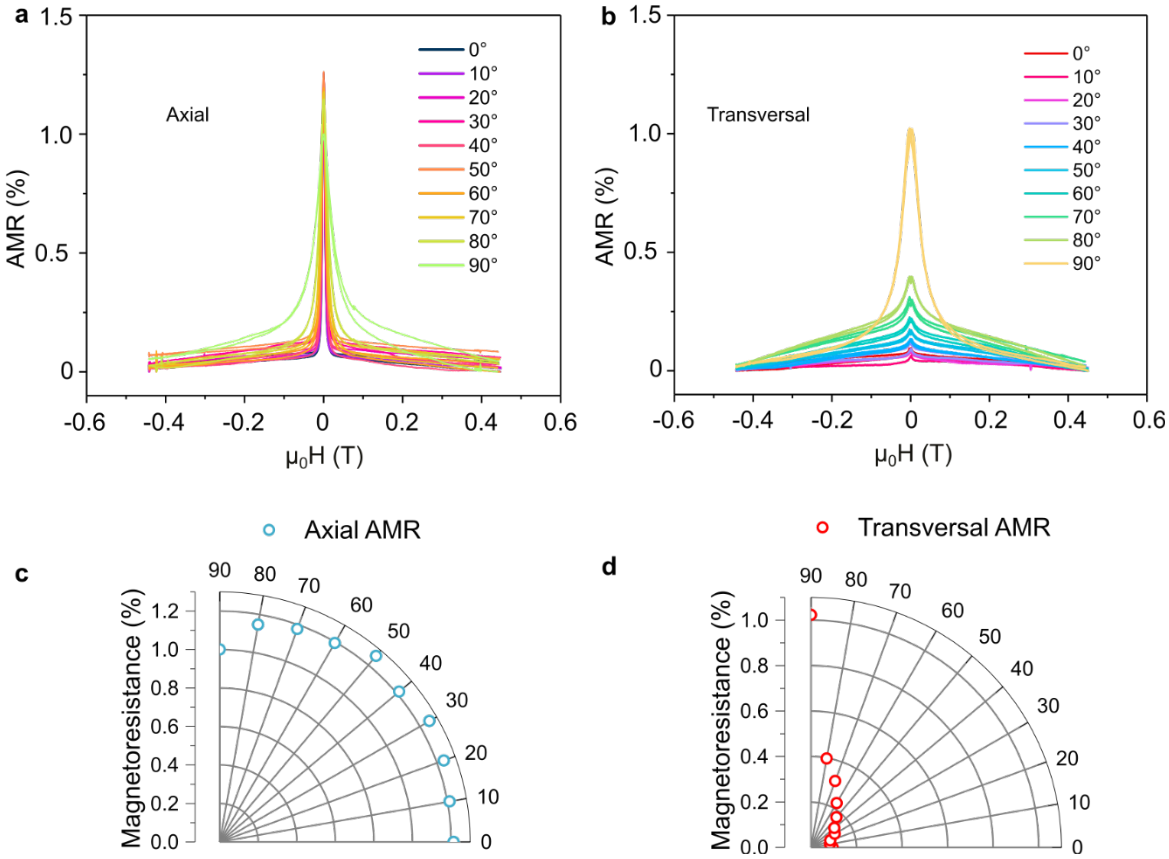

Supplementary Figure S18. Angular-dependent response of flexible AMR sensors. AMR responses of overbraided magnetoresistive sensors measured at different angular position of the sensor plane with respect to the magnetic field. AMR response of a overbraided sensor rotated (a) around the overbraided sensor's axis and (b) transversal to the overbraided sensor's axis. (c) The magnitude of the AMR effect remains comparable at rotations around the axis of the overbraided sensor but (d) drops upon rotations transversal to the axis in line with the anisotropic magnetoresistive effect.

Supplementary Videos

Supplementary Video 1. Overbraided magnetoresistive sensor before, during and after a mechanical interaction. The video showcases real-time measurements of a overbraided magnetoresistive sensor captured before, during, and after mechanical interactions (such as crumpling and touching) of the armband containing overbraided magnetoresistive sensors. Measurements of a single overbraided magnetoresistive sensor are shown. For the initial ≈30 s of the video, there was no magnet in proximity to the armband. Subsequently, a magnet was brought close to the overbraided sensor and maintained in position for ≈30 s. The magnet was then withdrawn for an additional ≈30 s. This sequence was repeated for the subsequent 60 s. Following this a hand was placed on top of the armband and pressure was applied on the armband for ≈30 s. Thereafter, the armband was crumbled for ≈30 s. Then the initial sequence of bring the magnet close to the armband for ≈30 s and taking it away for ≈30 s was repeated for 120 s. The functionality of the overbraided magnetoresistive sensor was not impacted by the mechanical interactions with the armband. Data were captured using a 12 bit ADC Adafruit HUZZAH ESP32 board and a voltage divider was used for interfacing with the sensors.

Supplementary Video 2. Magnetosensitive wristband in underwater conditions. A wristband integrated with a waterproof AMR overbraided magnetoresistive sensors is activated when used in underwater conditions. The video shows a user wearing the wristband below the water level in a plastic container. The wristband is connected to a Tensormeter measuring unit (HZDR Innovation GmbH, Germany) to detect the changes in resistance using a dedicated virtual instrument controlled via LabVIEW. The sensor is biased at 2 V and 1 mA during the measurement. When a permanent magnet is approached to the overbraided sensor from air until dipping under the water, the sensor is able to detect the proximity of the magnet in such harsh conditions via changes in magnetoresistance. The overbraided magnetoresistive sensors can be selectively activated without unwanted direct touch causing false activations (see movie at 14 s). These increased capabilities position this overbraided magnetoresistive sensor technology as a reliable interface that will be activated selectively upon target interaction with magnetic objects that will react in typical humid, sweat and rainy conditions like in sportwear and even in aquatic activities for underwater actuation. This opens a new area of application of smart interactive textiles in high performance garments that were previously not accessible due to artefacts caused by water like in capacitive interfaces.

Supplementary Video 3. Knitted armband interacting with a virtual reality (VR) environment. The video showcases real-time interaction using the armband in a VR environment. The armband is utilised to successfully navigate through the VR environment where two overbraided magnetoresistive sensors are used to move forward and turn left. In this video the wearer uses a magnet in a ring to navigate through the VR environment. For the first 5 s the wearer moves forward in the VR environment using one overbraided magnetoresistive sensor and then takes left turn using another overbraided magnetoresistive sensor. Thereafter, the wearer moves for another 5 s, before turning left again. Then moves forward again until taking a left turn. Data was captured using a 12 bit ADC Adafruit HUZZAH ESP32 board. Voltage dividers were used for interfacing with the sensors. The sensor measurements were sent over the OSC protocol to a Unity 3D application controlling the locomotion.

Supplementary Video 4. Magnetoresistive strap indicating if a helmet is secured on. The video demonstrates the functionality of the overbraided magnetoresistive sensor which is securely fastened onto a helmet. The overbraided sensor provides a signal to assess if the helmet is securely fastened or not. First, the helmet strap is securely fastened, which is reflected at the indicator attached to the helmet. Then the helmet strap is unfastened and fastened three times in succession. The indicator attached to the helmet indicates whether the strap is fastened or not. Towards the end of the video, the helmet strap is unfastened, and this action is displayed on the screen. A 12 bit ADC LilyGO ESP32-S3 board along with a voltage divider was utilised to measure the overbraided magnetoresistive sensor.

References

[1] D. Teichmann, A. Kuhn, S. Leonhardt, M. Walter, *Sensors* **2013**, *14*, 1.

[2] H. Dalkılıç, H. Özdemir, M. H. Özcanhan, *Textile Research Journal* **2024**, *0*,0.

[3] W. Zhang, Q. Guo, Y. Duan, C. Xing, Z. Peng, *IEEE Sensors Journal* **2022**, *22*,11.

[4] L. Ding, S. Xuan, J. Feng, X. Gong, *Composites Part A: Applied Science and Manufacturing* **2017**, 100.

[1] T. Hughes-Riley, P. Jobling, T. Dias, S. H. Faulkner, *Textile Research Journal* **2021**, *91*, 624.

[2] J. Park, S. H. Park, S. H. Jeong, J. Y. Lee, J. Y. Song, *Front. Chem.* **2023**, *11*, 1090648.

[3] G. Paul, R. Torah, K. Yang, S. Beeby, J. Tudor, *Meas. Sci. Technol.* **2014**, *25*, 025006.

[4] H. Shahariar, I. Kim, H. Soewardiman, J. S. Jur, *ACS Appl Mater Interfaces* **2019**, *11*, 6208.

[5] N. Abeywickrama, M. Kgatuke, K. Marasinghe, M. N. Nashed, C. Oliveira, A. M. Shahidi, T. Dias, T. Hughes-Riley, *Materials* **2023**, *16*, 4129.

[6] P. Lugoda, T. Hughes-Riley, R. Morris, T. Dias, P. Lugoda, T. Hughes-Riley, R. Morris, T. Dias, *Sensors* **2018**, *18*, 2369.

[7] M. N. Nashed, D. A. Hardy, T. Hughes-Riley, T. Dias, *Fibers* **2019**, *7*, 12.

[8] P. Lugoda, J. C. Costa, C. Oliveira, L. A. Garcia-Garcia, S. D. Wickramasinghe, A. Pouryazdan, D. Roggen, T. Dias, N. Münzenrieder, *Sensors* **2019**, *20*, 73.

[13] N. Yang, H. Wu, S. Wang, G. Yuan, J. Zhang, O. Sokolov, M. I. Bichurin, K. Wang, Y. Wang, *APL Mater.* **2021**, 9, 021123.

[14] B. A. Kaidarova, W. Liu, L. Swanepoel, A. Almansouri, N. R. Geraldi, C. M. Duarte, J. Kosel. *npj Flex. Electron.* **2021**, 5, 2.

[15] C. Becker, B. Bao, D. D. Karnaushenko, V. K. Bandari, B. Rivkin, Z. Li, M. Faghih, D. Karnaushenko, O. G. Schmidt. *Nat. Commun.* **2022**, 13, 2121.

[16] R. Xu, G. S. C. Bermúdez, O. V. Pylypovskyi, O. M. Volkov, E. S. O. Mata, Y. Zabila, R. Illing, P. Makushko, P. Milkin, L. Ionov, J. Fassbender, D. Makarov. *Nat. Commun.* **2022**, 13, 6587.

[17] Y. Chen, D. Zhao, J. Shao, Z. Fu, C. Wang, S. Wang, J. Du, M. Zhong, J. Duan, Y. Li, Z. Hu. *Rev. Sci. Instrum.* **2023**, 94, 045005.

[18] M. Ha, G. S. C. Bermúdez, T. Kosub, I. Mönch, Y. Zabila, E. S. O. Mata, R. Illing, Y. Wang, J. Fassbender, D. Makarov. *Adv. Mater.* **2021**, 33, 2005521.
