# Supplementary material for: Submersible touchless interactivity in conformable textiles enabled by highly selective overbraided magnetoresistive sensors
Source: Commun Eng. 2025 Feb 25;4:33. doi: 10.1038/s44172-025-00373-x (PMC11861257; doi:10.1038/s44172-025-00373-x)
Supplement: Supplementary file 3 — Description of Additional Supplementary Files [file 44172_2025_373_MOESM3_ESM.pdf]

Description of Additional Supplementary Files

File name: Supplementary Movie 1

Description: Overbraided magnetoresistive sensor before, during and after a mechanical interaction. The video showcases real-time measurements of a overbraided magnetoresistive sensor captured before, during, and after mechanical interactions (such as crumpling and touching) of the armband containing overbraided magnetoresistive sensors. Measurements of a single overbraided magnetoresistive sensor are shown. For the initial ≈30 s of the video, there was no magnet in proximity to the armband. Subsequently, a magnet was brought close to the overbraided sensor and maintained in position for ≈30 s. The magnet was then withdrawn for an additional ≈30 s. This sequence was repeated for the subsequent 60 s. Following this a hand was placed on top of the armband and pressure was applied on the armband for ≈30 s. Thereafter, the armband was crumbled for ≈30 s. Then the initial sequence of bring the magnet close to the armband for ≈30 s and taking it away for ≈30 s was repeated for 120 s. The functionality of the overbraided magnetoresistive sensor was not impacted by the mechanical interactions with the armband. Data were captured using an 12 bit ADC Adafruit HUZZAH ESP32 board and a voltage divider was used for interfacing with the sensors.

File name: Supplementary Movie 2

Description: Magnetosensitive wristband in underwater conditions. A wristband integrated with a waterproof AMR overbraided magnetoresistive sensors is activated when used in underwater conditions. The video shows a user wearing the wristband below the water level in a plastic container. The wristband is connected to a Tensormeter measuring unit (HZDR Innovation GmbH, Germany) to detect the changes in resistance using a dedicated virtual instrument controlled via LabVIEW. The sensor is biased at 2 V and 1 mA during the measurement. When a permanent magnet is approached to the overbraided sensor from air until dipping under the water, the sensor is able to detect the proximity of the magnet in such harsh conditions via changes in magnetoresistance. The overbraided magnetoresistive sensors can be selectively activated without unwanted direct touch causing false activations (see movie at 14 s). These increased capabilities position this overbraided magnetoresistive sensor technology as a reliable interface that will be activated selectively upon target interaction with magnetic objects that will react in typical humid, sweat and rainy conditions like in sportwear and even in aquatic activities for underwater actuation. This opens a new area of application of smart interactive textiles in high performance garments that were previously not accessible due to artefacts caused by water like in capacitive interfaces.

File name: Supplementary Movie 3

Description: **Knitted armband interacting with a virtual reality (VR) environment.** The video showcases real-time interaction using the armband in a VR environment. The armband is utilised to successfully navigate through the VR environment where two overbraided magnetoresistive sensors are used to move forward and turn left. In this video the wearer uses a magnet in a ring to navigate through the VR environment. For the first 5 s the wearer moves forward in the VR environment using one overbraided magnetoresistive sensor and then takes left turn using another overbraided magnetoresistive sensor. Thereafter, the wearer moves for another 5 s, before turning left again. Then moves forward again until taking a left turn. Data was captured using a 12 bit ADC Adafruit HUZZAH ESP32 board. Voltage dividers were used for interfacing with the sensors. The sensor measurements were sent over the OSC protocol to a Unity 3D application controlling the locomotion.

File name: Supplementary Movie 4

Description: **Magnetoresistive strap indicating if a helmet is secured on.** The video demonstrates the functionality of the overbraided magnetoresistive sensor which is securely fastened onto a helmet. The overbraided sensor provides a signal to assess if the helmet is securely fastened or not. First, the helmet strap is securely fastened, which is reflected at the indicator attached to the helmet. Then the helmet strap is unfastened and fastened three times in succession. The indicator attached to the helmet indicates whether the strap is fastened or not. Towards the end of the video, the helmet strap is unfastened, and this action is displayed on the screen. A 12 bit ADC LilyGO ESP32-S3 board along with a voltage divider was utilised to measure the overbraided magnetoresistive sensor.

File name: Supplementary Data 1

Description: **Source Data for Figures**
